# Effect of Bacteriophage Administration Route on Phage Localization in a Rat MRSA Implant-Associated Infection Model

**DOI:** 10.3390/antibiotics15070633

**Published:** 2026-06-23

**Authors:** Yusuf Hakan Abacı, Onur Genç, Erdem Ateş, Hatice Oruç Demirbağ, Cengiz Yılmaz

**Affiliations:** 1 Acıbadem Adana Hospital, 01130 Adana, Turkey; 2 Anamur State Hospital, 33640 Mersin, Turkey; onur.genc4@saglik.gov.tr; 3 Mersin City Education and Research Hospital, 33240 Mersin, Turkey; erdem.ates1@saglik.gov.tr; 4 Department of Histology and Embryology, Faculty of Medicine, Mersin University, 33110 Mersin, Turkey; horuc@mersin.edu.tr; 5 Department of Orthopedics and Traumatology, Faculty of Medicine, Mersin University, 33110 Mersin, Turkey; cyilmaz@mersin.edu.tr

**Keywords:** implant-associated infection, implant, bacteriophage therapy, animal experiment

## Abstract

**Background/Objectives:** Implant-associated infections are challenging conditions in orthopedic surgery. This experimental study aimed to evaluate phage localization within infected tissues following different routes of administration. **Methods:** An implant-related infection model was created using *methicillin-resistant Staphylococcus aureus* (MRSA) in twenty-four rats. Subjects were randomly divided into four groups depending on the bacteriophage administration route. Three rats were designated as the control group. Phage suspension was applied intraperitoneally, intravenously, orally and locally at 0.1 mL/day of 1 × 10^8^ PFU/mL suspension for three consecutive days. In the control group, intravenous, intraperitoneal and oral phage suspensions were administered separately at the same dose for 3 days. After completion of the experiment, tibia samples were taken in the experimental group. Additionally, liver, kidney, stomach, brain, heart muscle and striated muscle tissue samples were taken from the three subjects in the control group. **Results:** In the control group, unconfirmed phage-like structures were incidentally observed in some mitochondria of renal proximal tubular epithelial cells on transmission electron microscopy. In the experimental group, there was a strong positive linear relationship between the total number of bacteria and the number of bacteriophage clusters, independent of the groups. **Conclusions:** Bacteriophage clusters were detected in infected tibial tissues after all administration routes, suggesting phage localization at the infection site. Unexpected phage-like clusters were observed within mitochondria of proximal tubular epithelial cells in the control animals. This finding should be regarded as an unconfirmed incidental finding requiring further validation.

## 1. Introduction

After the discovery of bacteriophages in the early 20th century, many researchers investigated phages as possible therapeutic agents due to their potential to kill bacteria. Today, bacteriophages are receiving great attention for their potential to be used as antibacterials, phage display systems, and vaccine delivery vehicles [1,2]. In the last decade, the emergence of multidrug-resistant bacteria has prompted researchers to reconsider this age-old approach and take a fresh look at phage therapy as a “new” and potentially viable treatment option for difficult-to-treat bacterial pathogens [3].

To date, human phage therapy trials have remained largely experimental, and routine use of human phage therapy has been limited to Georgia, Poland, and Russia [4]. Most of the current phage availability is from the Eliava Institute of Bacteriophage, Microbiology and Virology, founded in Georgia in 1923 [5].

Since 2013, bacteriophages have been investigated experimentally in orthopedic implant-associated infection models. In a study by Yılmaz et al., the addition of bacteriophages to antibiotic therapy improved biofilm penetration compared with antibiotic or phage monotherapy [6].

Since most of the phage therapy studies are experimental, there is no consensus on the route and dose of administration for the phage containing suspensions. Oral, intraperitoneal injection, intravenous injection, and local injection options are available. This study aimed to evaluate whether different routes of administration influence the ultrastructural localization of bacteriophages within infected tissues. For this purpose a previously validated implant-associated infection animal model was used [7].

The aim of this study was to evaluate whether different routes of bacteriophage administration affect phage localization within infected tissues in a rat MRSA implant-associated infection model. We hypothesized that bacteriophages would successfully reach the infected area regardless of the administration route but that differences in phage localization patterns might be observed among delivery methods.

## 2. Results

**Figure 1:**
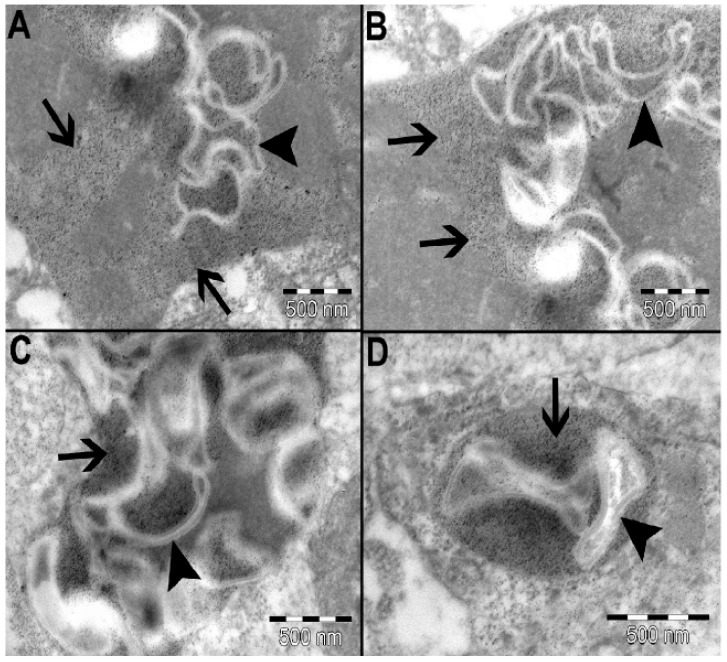
Representative transmission electron microscopy images obtained from the intraperitoneal (IP) administration group. Degenerated bacterial cells (arrowheads) and bacteriophage clusters (arrows) are demonstrated within the infected tibial tissue. (**A**) ×50,000, (**B**) ×50,000, (**C**) ×60,000, (**D**) ×75,000.

**Figure 2:**
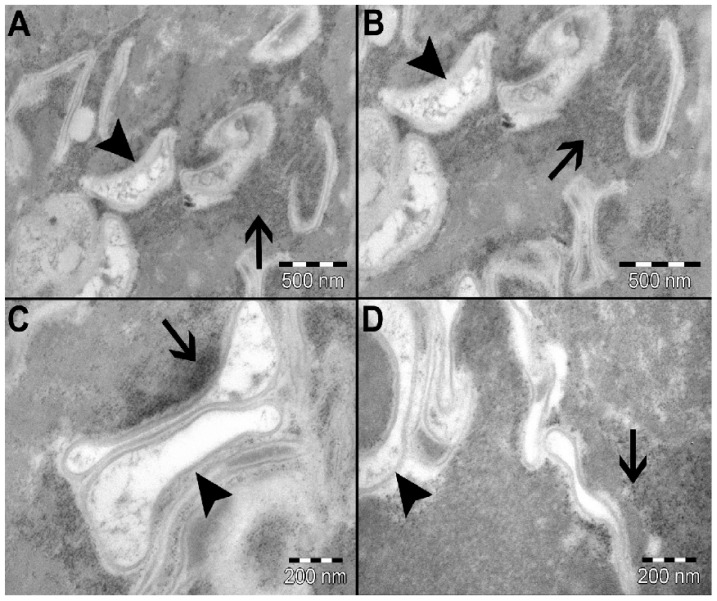
Representative transmission electron microscopy images from the local injection (LI) group showing degenerated bacterial cells (arrowheads) and bacteriophage clusters (arrows) within the infected tissue. (**A**) ×50,000, (**B**) ×60,000, (**C**) ×100,000, (**D**) ×100,000.

**Figure 3:**
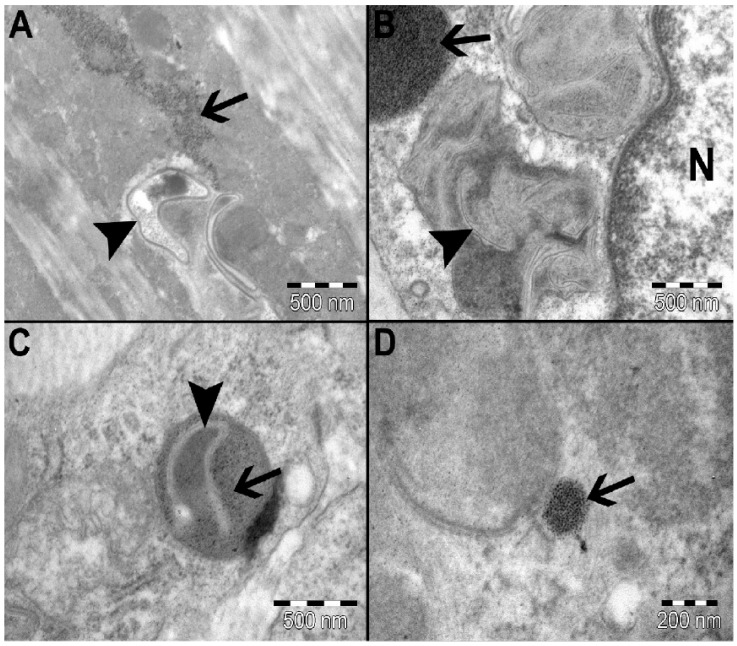
Representative transmission electron microscopy images from the oral administration (PO) group demonstrating bacterial degeneration (arrowheads) and associated bacteriophage clusters (arrows). (**A**) ×50,000, (**B**) ×50,000, (**C**) ×60,000, (**D**) ×100,000.

**Figure 4:**
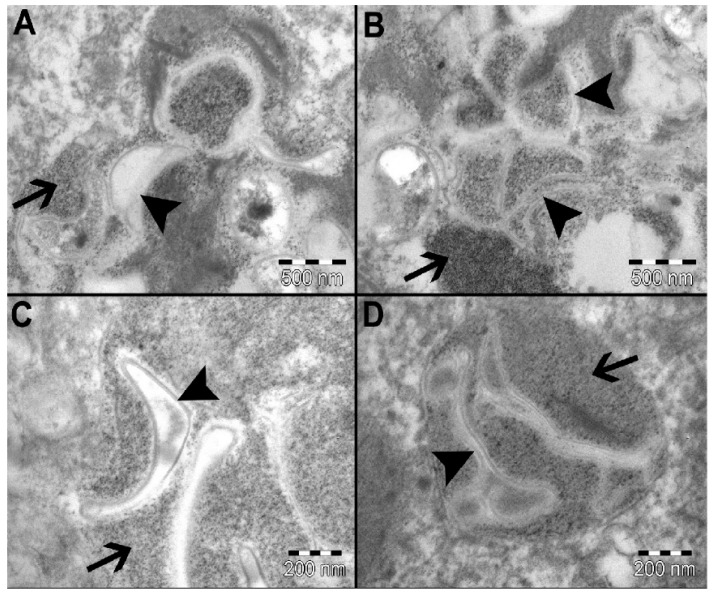
Representative transmission electron microscopy images from the intravenous (IV) group showing bacteriophage clusters (arrows) associated with degenerated bacterial cells (arrowheads) in infected tibial specimens. (**A**) ×50,000, (**B**) ×50,000, (**C**) ×100,000, (**D**) ×100,000.

Infection developed clinically in all subjects except in the control group. At the end of the 14-day infection period, animals in the infected groups exhibited an approximately 20% reduction in body weight compared with baseline measurements, supporting the establishment of clinically relevant infection. Microscopic examination of the subject groups revealed different densities of bacteria and bacteriophages in all subject samples (Figure 1, Figure 2, Figure 3 and Figure 4). Bacteria were observed to be either intact or phagocytosed by other cells. Total numbers of the bacteria counted in the scanned sample area were recorded. Bacteriophages were found in some bacteria. The bacteriophages could not be counted due to their size; instead, they were counted as clusters either within bacteria or as free clumps. The results of the analysis are given in Table 1.

Results were compared statistically, comparing all groups to each other. No statistically significant difference was found between the numbers of bacteria, numbers of phagocytosed phage-containing or non-phage-containing bacteria, and numbers of bacteriophage clusters (Table 2).

Independent of the groups, there was a strong positive linear relationship between the total number of bacteria and the number of bacteriophage clusters (r = 0.745; *p* < 0.001). However, this finding should be interpreted only as a morphological association and not as evidence of phage concentration, bacterial viability, phage replication, or therapeutic efficacy.

**Figure 5:**
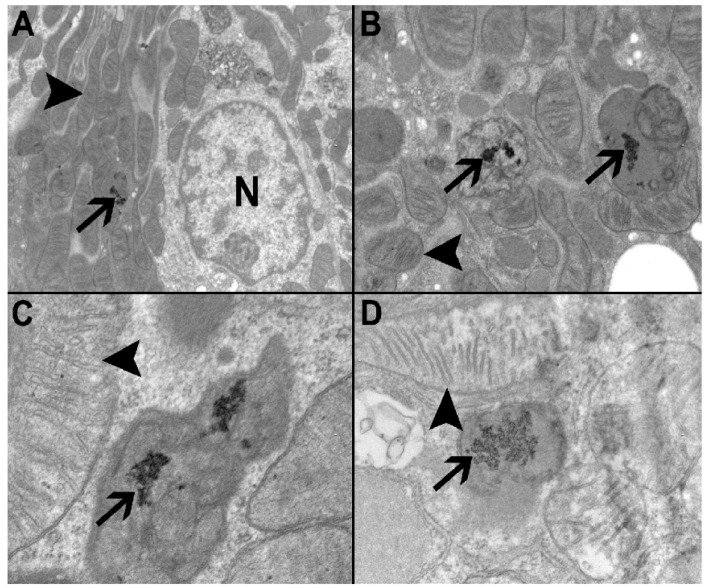
Phage-like structures (arrow) and mitochondria (arrowhead) images in the kidney proximal tubules of rats in the control group. (**A**) ×50,000, (**B**) ×50,000, (**C**) ×100,000, (**D**) ×100,000.

The control group was not inoculated with bacteria; instead, bacteriophages alone were administered via different routes. In the electron microscopic examination of this group of subject’s samples, no bacteriophages were observed in bone, liver, stomach, brain, heart muscle or striated muscle tissues. But, in all three subjects of the control group, whether administered intravenously, intraperitoneally or orally, unconfirmed phage-like structures were incidentally observed in some mitochondria of proximal tubular epithelial cells. These mitochondria showed ultrastructural alterations, including degeneration and loss of cristae (Figure 5).

## 3. Discussion

Although single-stage treatment has gained increasing attention in implant-associated infections, many patients still require complex treatment protocols involving prolonged antimicrobial therapy and multiple surgical procedures. The emergence of multidrug-resistant organisms further complicates treatment and may increase the risk of treatment failure and additional surgical interventions [8,9].

Bacteriophages have the ability to disrupt the extracellular matrix of the biofilm through the use of depolymerase enzymes [6,10]. In the study by Capparelli et al. on mice inoculated with Staphylococcus aureus, including MRSA, it has been reported that bacteriophages are 97% effective against infectious material given at a lethal dose and provide eradication of infection within 10 days in subjects with non-fatal infection. The reported high efficacy and low side-effect profile suggest that bacteriophages may offer potential advantages, particularly in patients with multi-organ failure who are at high risk of mortality [11].

The clinical use of bacteriophages commenced many years ago. In a study by Sakandelidze and Meĭpariani conducted in 1974, 236 patients with Staphylococcus, Streptococcus, and Proteus infections, including osteomyelitis, peritonitis, lung abscesses, and post-surgical wound infections, were treated with bacteriophages administered subcutaneously or through a surgical drain, and the researchers reported that they eliminated antibiotic-resistant infections in 92% of patients [12]. In 1977, Kvachadze isolated a particularly active phage from an Eliava Institute phage cocktail used to treat staphylococcal infections. The phage isolate was named Sb-1 [13,14]. Sb-1 is suitable for phage therapy, multiplies only by lytic infections, does not produce a lysogenic state and is therefore a lethal phage. Fish et al. successfully used the Sb-1 phage in the treatment of patients with diabetic finger ulcers infected with *S. aureus* [15]. The same agent was preferred in this study.

The implant-associated infection model carried out in the study is a model that has been successfully applied before in the literature [6,7]. Development of clinically visible infection in all subjects and the fact that there was no significant difference between the groups in terms of the total number of bacteria confirms that the establishment of infection was successful.

The administration method of phages also affects pharmacokinetics. Intramuscular, intraperitoneal, and subcutaneous injections of a phage cocktail were compared for efficacy in a murine burn model. It has been argued that intraperitoneal injection is the most effective way because it delivers more phages faster and longer. It has been suggested that the differences in the efficacy of these three different administration methods could be explained by the pharmacokinetics of phage delivery to the blood, spleen, and liver [16]. We investigated bacteriophage localization following different routes of administration and observed bacteriophage clusters within infected tissues in all treatment groups. However, because an infected, untreated control group was not included, these findings should not be interpreted as evidence of therapeutic efficacy.

Rather than administration route, number of bacteria at the infected site determined the number of phages. A strong positive relationship independent of groups was observed between the total number of bacteria and the number of bacteriophage clusters. Similarly, in a clinical study in which phage applications were tried in the treatment of chronic ear infections, it was observed that the phage concentration increased 200 times in the samples taken while the infection was ongoing and that the phages disappeared when the infection had been treated [17].

In electron microscopic examination of samples obtained from the control group subjects, regardless of administration method, an unexpected incidental observation was the presence of phage-like structures within some mitochondria of renal proximal tubular epithelial cells. The normal ultrastructure of these mitochondria was disrupted; degeneration and decrease were observed in normal mitochondrial crista. According to the endosymbiosis theory, it is argued that mitochondria originate from proteobacteria [18,19]. Mitochondria are the only self-replicating cellular organelles with their own genetic material. Its circular genetic material, lack of histone packaging, and presence of outer and inner mitochondrial membrane are some features that highlight its bacterial ancestry. Although mitochondria have largely lost their independence over time, it is noteworthy that some prokaryotic features persist in these organelles [20]. Based on the theory, the presence of phage-like structures within mitochondria may be considered a hypothesis-generating observation. Since proximal tubular epithelial cells are metabolically active and rich in mitochondria, this finding may warrant further investigation. However, in the absence of confirmatory methods, it should not be interpreted as evidence of mitochondrial affinity or phage-induced mitochondrial damage. The potential tissue effects of bacteriophages at various doses and under different conditions should be evaluated in separate studies. However the impression that bacteriophages are specific to bacteria and harmless in human tissue should be approached with caution.

Several limitations of the present study should be acknowledged. First, although animals were monitored daily for the development of clinical signs of infection and body weight measurements were recorded, body temperature and other quantitative clinical severity parameters were not systematically assessed. No mortality occurred during the study period; therefore, survival analyses were not performed. Second, the primary objective of this study was to investigate bacteriophage localization within infected tibial tissue rather than treatment efficacy. Accordingly, phage antibacterial activity and therapeutic outcomes were not evaluated. Phage burden and bacterial viability were not assessed by plaque assay or culture; therefore, TEM-based counts should be interpreted only as ultrastructural localization findings. Third, microbiological assessment of distant organs was not performed, and therefore the presence or absence of disseminated bacterial infection could not be determined. Similarly, organ biodistribution analyses were performed only in the non-infected control animals, as additional organ harvesting from infected animals was not included in the original study design. Fourth, transmission electron microscopy was selected as the primary imaging modality for ultrastructural evaluation. Consequently, complementary histological or immunohistochemical staining techniques that could further characterize host tissue responses and phage localization were not performed. Finally, the number of experimental animals was determined by a priori power analysis and kept at the minimum required level in accordance with ethical principles for animal experimentation. Future studies incorporating comprehensive clinical monitoring, systemic organ evaluation, complementary histological methods, and infected-animal biodistribution analyses may provide a more complete understanding of bacteriophage behavior in implant-associated infections.

An unexpected incidental finding of the present study was the observation of phage-like clusters within the mitochondria of proximal tubular epithelial cells in the kidneys of control animals. Because this observation was not anticipated during study planning, kidney tissue from infected animals was not examined. Further studies are required to confirm this finding and to determine its biological significance.

## 4. Materials and Methods

Institutional animal research ethics committee permission was obtained (Mersin University Animal Experiments Local Ethics Committee, approval number: 52602694-050.01.04-E-1583694; date of approval: 9 February 2021). Twenty-seven *Wistar Albino* rats, 12–14-week-old males (200–240 g), were used. The number of experimental animals was determined as a minimum of 24 by power analysis (type 1 error 0.05, test power 80% and effect size 0.816). The subjects were divided into 4 experimental groups of 6 animals in each and a separate control group of 3 rats. The implant-associated infection model was developed in the experimental groups. The PO group received oral phage therapy; the IP group received intraperitoneal injection of phages; the IV group received intravenous injection of phages; and the LI group received local injection of phages to the infected site. The control group animals did not undergo the implant-associated infection model procedure; instead, they only received the phage therapy. One of the administration routes was utilized for each one of the subjects in the control group. Local injection was not done because no infection was created in this group.

Throughout the study, the subjects were cared for at 24° ambient temperatures, 55–60% humidity, and a 12:12 h light–dark cycle, and free access to dry food and tap water was provided. The animal model described by Ersoz et al. was used to establish an implant-associated infection model with a *methicillin-resistant Staphylococcus aureus* (MRSA) strain [7].

### 4.1. Making of the Biofilm-Covered Infected Prosthetic Catheters

Plastic intravenous (iv) catheters infected with MRSA and having a biofilm layer were used to create this model. A MRSA strain was seeded on 5% blood agar (Merck, Darmstadt, Germany) for passage and incubated at 37 °C for 18 h. Two-to-three colonies of the growing strains were suspended in 5 cc of saline and centrifuged at 3000 rpm (revolution per minute) for 5 min; the supernatant was discarded, and the same procedure was repeated 2 more times. A suspension of 0.5 McFarland turbidity (10^8^ CFU/mL (coloni-forming units/milliliter)) was obtained and was diluted 10 times. Five hundred microliters of the diluted bacterial suspension was added to 2 mL of tryptic soy broth (TSB) containing 2% glucose. A sterile 0.5 cm long IV cannula was inserted into this solution containing 10^6^ CFU/mL of bacteria. The tubes containing the catheters were incubated at 37 °C for 18 h. The solution in the tubes was emptied under aseptic conditions, washed three times with 5 cc saline, and transferred at 4 degrees to be used in the experiment. For confirmation of pre-colonization, 5 pre-colonized cannulas were randomly selected and incubated on blood agar, and growth was checked semi-quantitatively. The number of colonies after 18 h of incubation was observed and verified.

### 4.2. Implant-Associated Infection Model

**Figure 6:**
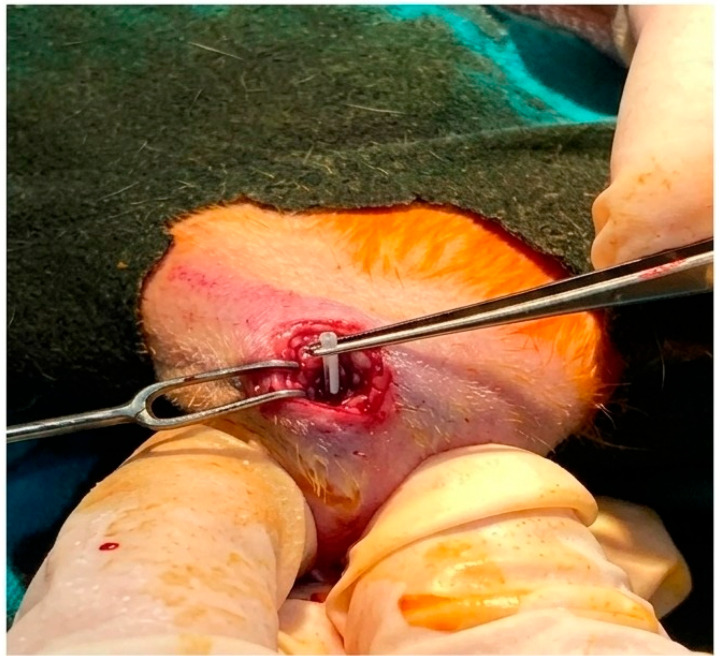
Insertion of MRSA-infected plastic catheter.

All of the experimental group subjects underwent the implant-associated infection model procedure. Ketamine and Xylazine (90–10 mg/kg respectively, 1cc) were administered intraperitoneally to 24 *Wistar Albino* rats with an average weight of 200–240 g. After local application of povidone to the surgical area, a mini incision was made to the right knee joints. The proximal articular surface of the tibia was exposed by preserving the patellar tendon. The intramedullary area was reached by drilling a hole in the bone just inferior to the proximal articular surface of the tibia. Sclerosing agent (5% sodium morrhuate, 0.15 mL volume) was injected into the bone cavity; 0.8 cm plastic intravenous catheters containing the MRSA strain and the biofilm were inserted into the tibial intramedullary areas of the rats (Figure 6). The incision was sutured. Subjects were observed daily for 14 days for signs of clinical infection. Body weight measurements were recorded at baseline and at the end of the 14-day infection period.

### 4.3. Bacteriophage Therapy

**Figure 7:**
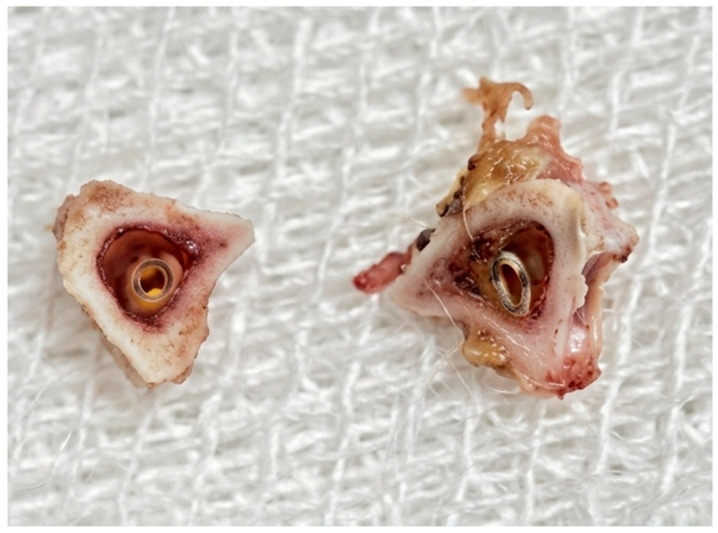
Example of tibial block with the infected intramedullary catheter.

After the completion of 14 days and confirmation of occurrence of clinical signs of infection such as erythema and draining sinus, phage therapy was administered to the subjects. The subjects were randomly divided into four groups, each containing 6 rats. According to the bacteriophage administration route, the groups were named as intraperitoneal (IP), intravenous (IV), oral (PO), and local injection (LI). Three rats formed the control group. Sb-1 (Eliava Institute, Microbiology and Virology, Tbilisi, Georgia) phage known to be effective against *Staphylococcus aureus* was used as the bacteriophage. Phage suspension (1 × 10^8^ PFU/mL suspension to 0.1 mL/day) was applied as intraperitoneal injection (IP group), intravenous injection into the jugular vein (IV group), oral (PO group) or surgical wound site local injection (LI group) for three days consecutively. In the control group, the same dose of intravenous, intraperitoneal and oral phage suspensions were administered separately to the subjects for 3 days. Because the jugular vein was used for injection, only the IV group received general anesthesia for 3 consecutive days during the phage therapy. Bacteriophage therapy was performed in all groups for three consecutive days. In this entire process, which included the creation of an infection model and the phage treatment phase, pain management was performed by applying acetaminophen at a dose of 1–2 mg/mL drinking water, after both the experiment and the treatments. The day after the completion of the treatment, the subjects were sacrificed by exsanguination under general anesthesia. Under sterile conditions, the right tibia of the rats was excised, and a 0.5 cm sample from the proximal tibial segment containing the infected catheter was removed with the help of a mini electric saw (Dremel 4000, 175 watts, Robert Bosch Tool Corporation, Mount Prospect, IL, USA.) (Figure 7). The removed bones were placed in glutaraldehyde-containing tubes and numbered. The histopathologic examination was blinded to the groups. In the control group, bone, liver, kidney, stomach, brain, cardiac muscle, and skeletal muscle tissue samples were obtained from all three animals.

### 4.4. Electron Microscopy Tissue Procedure

Tibial tissue samples taken for electron microscopic examinations were fixed in 2.5% glutaraldehyde solution for 4–6 h. The fixed tissues were decalcified using 0.15 M EDTA solution containing 1.9% glutaraldehyde for 3 weeks. After the decalcification process was completed, the tibiae were divided into four parts with the intravenous catheter inside and washed in buffered solution. Samples were divided into 1 mm^3^ pieces and subjected to the protocol listed below.
1.% 1 osmium tetraoxides1 h.2.Washing with PBS3 × 5 min.3.% 50 ethyl alcohol15 min (4 °C).4.% 60 ethyl alcohol15 min (4 °C).5.% 70 ethyl alcohol15 min (4 °C).6.% 80 ethyl alcohol15 min (4 °C).7.% 96 ethyl alcohol15 min (4 °C).8.% 100 ethyl alcohol15 min (4 °C).9.% 100 ethyl alcohol15 min (4 °C).10.% 100 ethyl alcohol15 min (25 °C).11.Propylene oxide15 min (25 °C).12.Propylene oxide15 min (25 °C).13.Propylene oxide + epoxy resin30 min (25 °C).14.Propylene oxide + epoxy resin30 min (25 °C).15.Epoxy resin16–24 h (25 °C).

The tissues were embedded in epoxy resin (Electron Microscopy Sciences^®®^ catalog no:13940, Hatfield, PA, USA) and polymerized in an oven—drying at 60 °C for 18 h. Next, 70-nanometer (nm)-thick sections were obtained from the blocks with an ultramicrotome (Leica^®®^ Ultracut UCT125, Leica Austria, Vienna) on 300-mesh copper grids. Thin sections were contrasted with uranyl acetate and lead citrate. The contrast procedure was as follows:
16.Uranyl acetate, 5 min.17.Washing with distilled water.18.Lead citrate, 5 min.19.Washing with distilled water.

Contrasted sections were analyzed with a transmission electron microscope (JEM-1011; JEOL Ltd., Tokyo, Japan) and photographed with a digital camera (Megaview III, Olympus GmbH, Münster, Germany). In electron microscopic examinations, the surface area of the tissue, which was first cut at small magnifications, was measured and recorded by the iTEM 5.0 (Olympus GmbH, Germany) image analysis program. On average, 0.19 mm^2^ of the surface area was examined for each subject. Bacteria and bacteriophage clusters were photographed and counted. The SPSS (Statistical Package for the Social Sciences) 25.0 package program was used in the statistical analysis of the data. The Shapiro–Wilk test was used to determine whether the parameters in the study showed normal distribution. The Kruskal–Wallis test was used to analyze the differences between the groups. In all tests, the statistical significance level was taken as 0.05.

## 5. Conclusions

In conclusion, bacteriophages were able to reach infected tissues in a rat MRSA implant-associated infection model regardless of the route of administration. No significant differences in phage localization were observed among the administration routes, whereas phage abundance was strongly associated with bacterial burden. Additionally, the observation of phage-like clusters within renal tubular mitochondria warrants further investigation. These findings contribute to our understanding of phage localization and may help guide future studies on bacteriophage therapy in implant-associated infections.

## Figures and Tables

**Table 1:** Scanned area and normalized counts per scanned area in electron microscopic analysis.

Group	Subject	Scanned Area (mm^2^)	Intact Bacteria (mm^2^)	Phagocytosed Phage-Free Bacteria (mm^2^)	Phagocytosed Phage-Containing Bacteria (mm^2^)	Free Phage Clusters (mm^2^)	Total Bacteria (mm^2^)
IP	1	0.14	0.0	592.9	3078.6	3235.7	3671.4
IP	2	0.30	16.7	2073.3	2220.0	2510.0	4310.0
IP	3	0.02	50.0	500.0	4000.0	4100.0	4550.0
IP	4	0.12	491.7	1625.0	0.0	66.7	2116.7
IP	5	0.10	0.0	6590.0	620.0	800.0	7210.0
IP	6	0.08	312.5	8637.5	4875.0	5587.5	13,825.0
LI	7	0.10	300.0	2300.0	2180.0	2500.0	4780.0
LI	8	0.09	33.3	2300.0	111.1	288.9	2444.4
LI	9	0.27	63.0	185.2	2051.9	2181.5	2300.0
LI	10	0.11	90.9	3445.5	181.8	181.8	3718.2
LI	11	0.24	8.3	79.2	916.7	1375.0	1004.2
LI	12	0.09	1322.2	5500.0	3177.8	3200.0	10,000.0
PO	13	0.10	0.0	320.0	400.0	470.0	720.0
PO	14	0.21	28.6	2123.8	309.5	471.4	2461.9
PO	15	0.29	17.2	2720.7	482.8	506.9	3220.7
PO	16	0.20	5.0	425.0	250.0	275.0	680.0
PO	17	0.33	3.0	0.0	1636.4	2200.0	1639.4
PO	18	0.24	100.0	1000.0	208.3	258.3	1308.3
IV	19	0.09	144.4	2133.3	1266.7	1700.0	3544.4
IV	20	0.44	2.3	81.8	13.6	0.0	84.1
IV	21	0.36	66.7	1302.8	305.6	347.2	1675.0
IV	22	0.31	3.2	87.1	12.9	0.0	90.3
IV	23	0.13	15.4	38.5	0.0	61.5	53.8
IV	24	0.10	350.0	5100.0	2660.0	3050.0	8110.0

Data are normalized by dividing each count by the corresponding scanned area. IP: intraperitoneal injection; LI: local injection; PO: oral administration; IV: intravenous injection; mm^2^: square millimeter.

**Table 2:** Comparison of normalized electron microscopic analysis results.

Parameter	Intraperitoneal Injection Median (IQR)	Local Injection Median (IQR)	Oral Administration Median (IQR)	Intravenous Injection Median (IQR)	*p* Value
Intact bacteria (mm^2^)	33.3 (4.2–246.9)	76.9 (40.7–247.7)	11.1 (3.5–25.7)	41.0 (6.3–125.0)	0.394
Phagocytosed phage-free bacteria (mm^2^)	1849.2 (850.9–5460.8)	2300.0 (713.9–3159.1)	712.5 (346.2–1842.9)	694.9 (83.1–1925.7)	0.409
Phagocytosed phage-containing bacteria (mm^2^)	2649.3 (1020.0–3769.6)	1484.3 (365.5–2148.0)	354.8 (264.9–462.1)	159.6 (13.1–1026.4)	0.204
Number of free phage clusters (mm^2^)	2872.9 (1227.5–3883.9)	1778.2 (560.4–2420.4)	470.7 (323.8–498.0)	204.4 (15.4–1361.8)	0.126
Total number of bacteria (mm^2^)	4430.0 (3831.1–6545.0)	3081.3 (2336.1–4514.5)	1473.9 (867.1–2256.3)	882.7 (85.6–3077.1)	* 0.047

Values are presented as median (interquartile range, Q1–Q3) after normalization to the scanned area (mm^2^). Between-group comparisons were performed using the Kruskal–Wallis test. * *p* < 0.05.

## Data Availability

The original contributions presented in this study are included in the article. Further inquiries can be directed to the corresponding author.

## Abbreviations

The following abbreviations are used in this manuscript:

MRSA	*methicillin-resistant Staphylococcus aureus*
mL	milliliter
PFU	plaque-forming unit
IP	intraperitoneal
IV	intravenous
LI	local injection
Rpm	revolution per minute
CFU	colony-forming unit
TSB	tryptic soy broth
mg	milligram
cc	cubic centimeter
cm	centimeter
nm	nanometer
mm^3^	cubic millimeter
°C	degree Celsius
SPSS	Statistical Package for the Social Sciences
